# The Host Range of *Fusarium proliferatum* in Western Canada

**DOI:** 10.3390/pathogens13050407

**Published:** 2024-05-14

**Authors:** Haitian Yu, Sheau-Fang Hwang, Stephen E. Strelkov

**Affiliations:** 1 Department of Agricultural, Food and Nutritional Science, University of Alberta, Edmonton, AB T6G 2P5, Canada; haitian7@ualberta.ca; 2 Institute of Food Crops, Yunnan Academy of Agricultural Science, Kunming 650205, China

**Keywords:** disease severity, *Fusarium proliferatum*, growth parameters, host range, principal component analysis, resistance, root rot

## Abstract

*Fusarium proliferatum* is associated with the root rot of many plant species, but knowledge of its impact on western Canadian field crops is limited. This study assessed the host range of this fungus and its effect on plant emergence, plant height, and shoot and root dry weights in repeated greenhouse experiments with wheat, barley, faba beans, peas, lentils, canola, lupine, and soybeans. Infection was confirmed via PCR, and principal component analysis determined the utility of different parameters in assessing host responses. All crops were at least partly susceptible, developing mild to severe disease at the seedling and adult stages, and showing significant reductions in growth. In general, the barley and wheat demonstrated higher tolerances to infection, followed by the faba bean and the pea. The soybean, canola, lupine, and lentil were most susceptible. The canola and the soybean were particularly vulnerable to *F. proliferatum* at the pre-emergence stage, while infection greatly reduced the lentil’s biomass. Reductions in the barley’s emergence and other growth parameters, however, occurred only under a high inoculum concentration. Variability in root rot severity among cultivars of the same crop indicated some diversity in host reactions within species. Nonetheless, the absence of fully-resistant crops may pose challenges in managing *F. proliferatum* in western Canadian cropping systems.

## 1. Introduction

Root rot is a destructive disease affecting many crops worldwide. It is caused by a variety of pathogens collectively known as the root rot complex, which includes, among others, *Rhizoctonia* spp., *Fusarium* spp., *Pythium* spp., and *Aphanomyces* spp. [1]. Infection by these pathogens typically results in reduced plant growth and the impaired biological function of the affected organs, leading to symptoms such as the rotting, wilting, yellowing, and discoloration of plant tissues [2].

Among these causal agents, the genus *Fusarium* includes widely distributed pathogenic fungi capable of infecting a broad range of plants [3]. Within this genus, numerous species have been found to cause root and crown rot in a variety of host crops. *Fusarium proliferatum*, in particular, has been increasingly reported to infect various hosts, including field, fruit, and vegetable crops [4,5,6]. The fungus has been identified as a causal agent of tissue rot and wilt diseases in many crops worldwide, including (among others) carnation (*Dianthus caryophyllus* L.) [7], cauliflower (*Brassica oleracea* var. *botrytis* L.) [8], garlic (*Allium sativum* L.) [9,10,11], onion (*Allium cepa* L.), maize (*Zea mays* subsp. *mays*), rice (*Oryza sativa* L.), and sugarcane (*Saccharum officinarum* L.) [12]. *Fusarium proliferatum* is also regarded as an important pathogen in the fruit industry, causing significant economic losses in bananas [13] and red-fleshed dragon fruit [14]. In Canada, *F. proliferatum* has been reported to cause crown and stem rot and pith necrosis in greenhouse-grown cannabis (*Cannabis sativa* L.) [15], as well as the root rot of soybeans (*Glycine max* L.) [16], with similar reports from the United States [17]. The fungus is also recognized as a threat to food and feed quality due to the production of mycotoxins such as fumonisins, which can adversely affect the sphingolipid metabolism, leading to chronic and acute diseases in humans and other animals [18].

Although it has been increasingly identified as a causal agent of tissue rot and wilting, the host range of *F. proliferatum* has been less studied than that of other *Fusarium* spp., creating a gap in the understanding of this genus; conversely, knowledge of the host range of plant pathogens is critical for effective disease management [19]. In the context of soilborne pathogens, host range often guides the choice of crops included in a rotation for disease mitigation. The inclusion of non-host crops can decrease the risk of disease development [20], and implementing diverse crop rotations may contribute to reducing pathogen populations in the soil [21]. On the other hand, the selection of susceptible or inappropriate hosts can result in an increase in inoculum levels and more severe disease development. Moreover, anticipated increases in root-related diseases, attributed to climate change [22], coupled with the widespread occurrence of pathogenic soilborne agents in agricultural systems [23], underscores the need for improved knowledge of the virulence of microbial pathogens and their capacity to cause diseases in different hosts. Such knowledge is essential for reducing losses from root rot and other diseases.

In the prairie region of western Canada, *Fusarium* spp. have been identified as the predominant pathogens responsible for root rot in multiple crops [24,25,26]. Among the most prevalent species of *Fusarium*, *F. avenaceum* has demonstrated aggressiveness on legumes, cereals, and canola [24,27,28]. Recently, *F. proliferatum* has been reported to cause severe reductions in the emergence and yield of canola under both field and controlled conditions [29]. To our knowledge, however, there are no reports on the host range of *F. proliferatum* or its impact on the plant germination or growth of other field crops in this region. The aim of this study was to investigate the reactions of eight important field crops grown in western Canada, including wheat (*Triticum aestivum* L.), barley (*Hordeum vulgare* L.), faba beans (*Vicia faba* L.), peas (*Pisum sativum* L.), lentils (*Lens culinaris* L. ssp. *culinaris*), canola (*Brassica napus* L.), lupine (*Lupinus angustifolius* L.), and soybeans (*Glycine max* L.), to inoculation with *F. proliferatum*. The specific objectives were as follows: (1) to assess the severity of the root rot caused by *F. proliferatum* in these crops; (2) to investigate the impact of *F. proliferatum* on emergence and plant growth in these crops; (3) to analyze the variation in responses to *F. proliferatum* among cultivars of the same crop; (4) to assess whether detrimental effects of infection persist to maturity; and (5) to evaluate the utility of assessments of disease severity, emergence, and plant growth parameters in determining the host range of this fungus. The results of this research may offer insights for formulating effective strategies to control root rot.

## 2. Materials and Methods

### 2.1. The Pathogen Material

Twenty isolates of *F. proliferatum*, originally collected from canola plants exhibiting symptoms of root rot and stored in the Applied Plant Pathology Lab of the University of Alberta [26], underwent a preliminary assessment to determine their aggressiveness on the canola cv. ‘Westar’. From these tests, isolate P002 emerged as being the most aggressive, based on its capacity to cause severe root rot and reduce plant height and biomass. Hence, isolate P002 was selected for use in this study. Fungal inoculum was generated on a wheat grain medium as described previously [30]. Briefly, 1 L of grain was soaked in tap water overnight at room temperature, and then transferred to a Hi-Patch mushroom spawn bag (Western Biologicals, Aldergrove, BC, Canada). The opening on the bag was closed with a foam insert and secured with a collar, and the grain was autoclaved for 90 min. After it cooled to room temperature, ~250 plugs (0.5-diam.) from a 14-days-old *F. proliferatum* culture grown on potato dextrose agar (PDA) were added, and the grain was thoroughly mixed. It was then incubated for 5 weeks in darkness at room temperature, allowing for the complete fungal colonization of the grain. The inoculated grain was air-dried for 3 days at 25 °C, ground to a powder (with a particle size ranging from 1–2 mm) and passed through a 2.0-mm-mesh. The ground grain inoculum was then stored in a cooler at 4 °C for a maximum of 2 months until use.

### 2.2. The Host Reaction at the Seedling Stage

The responses of eight crop species to *F. proliferatum* inoculation were assessed under greenhouse conditions at the seedling stage. The crops evaluated included wheats (‘Katepwa’, ‘AC Crystal’, and ‘Lillian’), barleys (‘AB Tofield’ and ‘Canmore’), faba beans (‘Malik’ and ‘Fabelle’), peas (‘CDC Greenwater’, ‘AAC Carver’, ‘CDC Amarillo’, and ‘AAC Barrhead’), lentils (‘CDC Nimble’ and ‘CDC Lima CL’), canolas (‘Westar’ and ‘L255PC’), lupines (‘Arabella’ and ‘Mirabor’), and soybeans (‘AAC Mandor’, ‘OT15-02’, and ‘AKRAS R2’). Briefly, a grain inoculum was mixed with a Promix PGX potting medium (Sun Gro Canada Inc., Seba Beach, AB, Canada) at grain inoculum-to-potting medium ratios of 1:300 (v:v) (0.33%, ‘low inoculum’ concentration) or 1:150 (v:v) (0.67%, ‘high inoculum’ concentration), corresponding to 3 × 10^4^ or 6 × 10^4^ colony-forming units (cfu) per g of potting medium, respectively. These inoculum levels were based on an earlier study [29]. Each host variety was sown in 473 mL cups (Uline, Toronto, ON, Canada) filled with 400 mL of the inoculated potting medium at a density of 10 seeds per cup and was maintained under greenhouse conditions at approximately 25 °C, with a 12 h photoperiod and supplemental light intensity ranging from 250 to 450 µmol/m^2^/s. The plants were watered as needed and fertilized (15N-15P-15K) weekly. Control treatments consisted of seeds grown in a non-inoculated potting medium. The experiment was arranged in a randomized complete block design (RCBD) with five replicates (cups) per treatment, and the entire experiment was repeated.

At 21 days after seeding, the plants were removed from the potting medium, and the roots were washed thoroughly with tap water to assess the root rot severity as described below in Section 2.4. After the completion of the disease assessment, the plants were dried for 7 days at ca. 25 °C, the shoots and roots were separated via cutting, and the respective dry weights were measured for each replicate (cup).

### 2.3. The Root Rot Development at Maturity

To evaluate the host reactions to *F. proliferatum* at the adult plant stage, greenhouse trials were conducted with one cultivar each of a canola (‘Westar’), faba bean (‘Fabelle’), soybean (‘AKRAS R2’), lupine (‘Arabella’), barley (‘Canmore’) and wheat (‘AC Crystal’), and three cultivars of peas (‘CDC Greenwater’, ‘AAC Carver’, ‘CDC Amarillo’). The plants were sown at a density of 10 seeds per cup and grown in 400 mL of a Promix PGX potting medium (Sun Gro Canada Inc., Seba Beach, AB, Canada). The potting medium was inoculated with grain inoculum at a ratio of 1:300 (v:v), equivalent to 3 × 10^4^ cfu per g of potting medium, at the time of seeding, as indicated above. Control treatments did not receive any grain inoculum, the experiment was arranged in an RCBD with three replicates (cups) per treatment, and the entire experiment was repeated.

After all the plants reached the flowering stage, they were carefully removed from the potting medium, and their roots were washed with tap water. Disease severity was then assessed according to the method described in Section 2.4. Diseased root tissue samples of the faba bean ‘Fabelle’, the soybean ‘AKRAS R2’, the lupine ‘Arabella’, the wheat ‘AC Crystal’ and the pea cultivars ‘CDC Greenwater’ and ‘AAC Carver’ were collected, flash-frozen in liquid nitrogen, and stored at −20 °C prior to DNA extraction. Before storage, all root samples underwent washing with tap water to remove any residual potting medium or visible debris. Subsequently, the samples were sterilized: the superficial layer (epidermis and cortex) of the diseased roots was excised, leaving only the interior vascular tissues. Tissues showing discoloration were then immersed in a 1% sodium hypochlorite solution for 1–2 min and rinsed three times in sterile distilled water.

### 2.4. Disease Ratings

Root rot severity was assessed on a 0–4 scale [25], illustrated for each crop in Supplementary Figure S1, where 0 = healthy roots; 1 = small, light-brown lesions were present on <25% of the tap root; 2 = brown lesions were present on 25–49% of the tap root; 3 = brown lesions were present on 50–74% of the tap root, with the tap root constricted; and 4 = the tap root was severely girdled, and there were brown lesions on >75% of the tap root with limited lateral roots. The final disease severity per experimental unit (cup) was calculated by averaging the values of all the individual plants within each cup.

### 2.5. Emergence, Plant Height, Shoot and Root Dry Weight

Emergence was determined by counting all surviving plants in each experimental unit 7 days after seeding. Plant height was measured from the soil line to the shoot apex with a ruler at 14 days after seeding. The shoot and root dry weights per each experimental unit were determined separately on a weighing scale (Fisher Science Education SLF 303, Thermo Fisher Scientific, Mississauga, ON, USA). Reductions in the seedling emergence, plant height, and shoot and root dry weights were calculated relative to those of the non-inoculated controls according to the equation:

Reduction = [(D_ck_ − D_tr_)/D_ck_] × 100%, (1)

where D_ck_ represents the control (non-inoculated) treatment and D_tr_ represents the inoculated treatment.

### 2.6. The PCR Detection of F. proliferatum 

The presence of fungal DNA in host tissues was determined via a PCR analysis with the *F. proliferatum*-specific primers TH5-F and TH6-R [31]. The specificity of the primers was confirmed by testing them on the *F. proliferatum* isolate P002 and isolates of *F. avenaceum*, *Fusarium graminearum*, *Fusarium solani*, *Fusarium redolens*, and *Fusarium oxysporum* obtained from the culture collection of the Applied Plant Pathology Lab, University of Alberta [26]. Fungal DNA was extracted from pure PDA-grown cultures using a DNeasy Plant Pro Kit (Qiagen, Hilden, Germany) according to the Quick-Start protocol (http://www.qiagen.com/HB-2552) (accessed on 25 May 2022). Extractions of total genomic DNA from inoculated and control plant roots were conducted using the same protocol and kit from 0.1 g of tissue. The quantity and quality of the DNA samples was assessed in a NanoDrop 2000 spectrophotometer (Thermo Fisher Scientific), and the final concentration of the DNA was adjusted to 20 ng/μL with nuclease-free water (Thermo Fisher Scientific). The samples were stored at −20 °C until needed.

PCR was conducted in a 20 μL reaction volume, which included 1 μL of the DNA template (20 ng/μL), 1 μL of each of the forward and reverse primers TH5-F and TH6-R (10 μM), 10 μL of HotStarTaq master mix (Qiagen), 0.25 μL of bovine serum albumin (10 mg/mL) (Thermo Fisher Scientific), and 6.75 μL of nuclease-free water (Thermo Fisher Scientific). Amplification reactions consisted of an initial heat activation at 95 °C for 10 min, followed by 40 cycles of denaturation at 94 °C for 1 min, annealing at 60 °C for 30 s, and an extension at 72 °C for 1 min. A final extension step was conducted at 72 °C for 5 min. Positive and negative controls (*F. proliferatum* DNA or an equivalent volume of nuclease-free water, respectively) were included in all PCR assays. Amplicons were resolved via agarose gel electrophoresis and the presence of a single band near the predicted amplicon size (~330 bp) was regarded as a positive result for *F. proliferatum* in the sample tested.

### 2.7. Data Analysis and Principal Component Analysis

All data sets were evaluated for homogeneity via an ANOVA with R Studio [32]. Unless otherwise noted, differences were considered significant at *p* < 0.05. If a significant interaction was detected between repetition and treatment, the data were analyzed separately to account for the effects of these factors on the results. Principal component analysis (PCA) was utilized to group the cultivars/crops into clusters based on their responses to *F. proliferatum* infection at the seedling stage, and to identify the primary parameters for evaluating host susceptibility. Root rot severity and reductions in seedling emergence, plant height, root and shoot dry weight were used to generate the PCA biplot.

## 3. Results

To evaluate the response of different crops to *F. proliferatum* infection, various parameters including disease severities, seedling emergences, plant heights, and shoot and root dry weights were recorded at the seedling stage. Disease severity was also assessed at maturity.

### 3.1. Root Rot Development at the Seedling Stage

The variance in emergence, plant height, and root dry weight was not significant (*p* > 0.05) in the two repeats of the experiment conducted at the seedling stage, while significant differences in the root rot severity and shoot dry weights were observed. Interactions between repeat and cultivar or crop and inoculum concentrations were not found to be significant, indicating consistent trends in the results. Due to their overall similarity and absence of significant interactions, the datasets from the two repeated experiments were combined for further statistical analysis.

All the crop species developed symptoms of root rot. Between 2–4 cultivars were examined per crop, and while some variability in disease severity was observed among cultivars of the same crop, none were completely resistant (Supplementary Table S1). When averaged across cultivars, disease severity ranged from 1.29 to 3.03 for each of the crops at the low inoculum concentration, and from 1.98 to 3.42 at the high inoculum concentration (Table 1). Root rot was significantly more severe at the high vs. the low inoculum concentration for all the species. When comparing the disease severity across the eight crops, the wheat and the barley generally developed the mildest symptoms at both inoculum concentrations, followed by the faba beans, lentils and peas (Table 1). At the lower inoculum concentration, there were no significant differences in root rot severity among the latter three crops, while at the higher concentration, disease was more severe in lentils vs. faba beans or peas. Root rot development tended to be most severe in canola, lupine, and soybeans, although at the high inoculum concentration, disease severity in these crops was similar to that of the lentils (Table 1). As expected, no disease symptoms were observed in any of the control (non-inoculated) treatments.

### 3.2. Emergence, Plant Height, Shoot and Root Dry Weights at the Seedling Stage

Inoculation with the low concentration of *F. proliferatum* did not affect the plant heights, emergences, or shoot and root dry weights in the barley; in this crop, only the high inoculum treatment significantly reduced these parameters relative to those of the control (Table 2). Similarly, no reductions in plant height and shoot dry weight were observed for the wheat at the low inoculum concentration, although significant reductions were found in the emergence and root dry weight in this treatment. At the high inoculum concentration, the plant height was significantly reduced in the wheat relative to the control, while the shoot dry weight declined compared with that in the low inoculum concentration. The emergence and root dry weight of wheat in the high inoculum treatment declined further compared with that of the low inoculum treatment (Table 2). In the faba beans, seedling emergence declined relative to the control under the high but not the low inoculum concentrations, while treatment with either concentration resulted in significant declines in height and shoot and root dry weights. In the cases of all the other crop species, treatment with either the low or the high *F. proliferatum* inoculum concentrations resulted in significant decreases in heights, emergences, and shoot and root dry weights relative to their respective controls, with these declines usually being more pronounced in the high inoculum treatment (Table 2). The reductions in plant heights, seedling emergences, and shoot and root dry weights for each of the 2–4 cultivars evaluated per crop species are included in Supplementary Figure S2.

### 3.3. Host Reaction at Maturity

Based on the ANOVA, the variance in the root rot severity between the two repeats of the experiment conducted at the adult plant stage was not significant, while significant differences were observed among hosts. Therefore, the disease severity data from the two repeats were combined to conduct a comparison among crops/cultivars.

At maturity, all crops presented symptoms of root rot, with disease severities ranging from 1.08 to 3.57 (Table 3). As was observed at the seedling stage, the lowest disease severity (1.08–1.15) at maturity was found on the barley and wheat, followed by the faba bean (2.00), one of the pea cultivars (‘ACC Carver’, 2.62), and the canola, soybeans, and the other two pea cultivars (3.00–3.26). The most severe root rot developed on lupine (disease severity = 3.57). PCR analysis with *F. proliferatum*-specific primers confirmed the presence of a single band of 300–400 bp (corresponding to the expected ~330 bp amplicon), which was obtained only from symptomatic plant tissues (Supplementary Figure S3).

### 3.4. Principal Component Analysis

**Figure 1:**
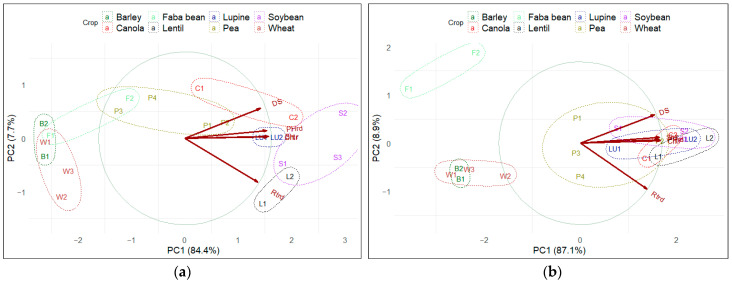
Principal component analysis based on root rot disease severity (DS) and reductions in the seedling emergences (Ctrd), plant heights (PHrd), shoot dry weights (Shrd), and root dry weights (Rtrd) of 20 cultivars representing 8 crop species grown in a potting medium treated with a low (**a**) or high (**b**) concentration of *Fusarium proliferatum* (3 × 10^4^ and 6 × 10^4^ colony-forming units/g potting medium, respectively). Disease severity and shoot and root dry weights were assessed at 21 days after seeding, while emergence was measured at 7 days and plant height was measured at 14 days after seeding. B1, barley cultivar ‘AB Tofield’; B2, barley ‘Canmore’; W1, wheat ‘Katepwa’; W2, wheat ‘AC Crystal’; W3, wheat ‘Lillian’; P1, pea ‘CDC Greenwater’; P2, pea ‘AAC Carver’; P3, pea ‘CDC Amarillo’; P4, pea ‘AAC Barrhead’; S1, soybean ‘AAC Mandor’; S2, soybean ‘OT15-02’; S3, soybean ‘AKRAS R2’; LU1, lupine ‘Arabella’; LU2, lupine ‘Mirabor’; L1, lentil ‘CDC Nimble’; L2, lentil ‘CDC Lima CL’; F1, faba bean ‘Malik’; F2, faba bean ‘Fabelle’; C1, canola ‘Westar’; C2, canola ‘L255PC’. Each crop is represented in a different color and circled with a dotted line. The red arrows represent the five parameters (DS, Ctrd, PHrd, Shrd, and Rtrd) used in PCA plot construction. The positions of these arrows close to the edge of the central circle indicate that all parameters were well represented in the analysis. Cultivars located along the direction of the arrow have high corresponding values, while those positioned in the opposite direction have low corresponding values.

Principal component analysis (PCA) was conducted on the root rot severity and reductions in the plant heights, seedling emergences, and shoot and root dry weights of all individual cultivars of each species at the seedling stage under the low and high *F. proliferatum* inoculum concentrations. For both inoculum concentrations, the five principal components collectively explained 100% of the variance among cultivars. The first principal component (PC1) explained 84.4% of the variance among cultivars at the low inoculum concentration, and 87.1% of the variance at the high inoculum concentration (Figure 1). The second principal component (PC2) explained 7.7% and 8.9% of the variance at the low and high inoculum concentrations, respectively. The third, fourth, and fifth principal components explained 5.4%, 2.2%, and 0.3% of the variation at the low inoculum concentration, and 1.9%, 1.4%, and 0.7% at the high inoculum concentration, respectively (Figure 1). The five parameters (the root rot severity and reductions in plant heights, seedling emergences, and shoot and root dry weights) collectively contributed to the most significant variation among all cultivars in PC1, with relative contributions ranging from −0.471 to −0.393 (Supplementary Table S2). The negative values indicated that these parameters were inversely related to the variation observed in PC1, implying that as disease severity increased or reductions in plant growth parameters became more pronounced, the values along PC1 decreased. The root rot severity and the reduction in root dry weights represented the most significant components in the second principal component (PC2), with relative contributions being over 0.8 and <−0.5, respectively (Supplementary Table S2). The broad distribution of the cultivars along the PC1 and PC2 axes of the biplots indicated significant variation among the cultivars of some crops, including faba beans and peas at both the high and low inoculum concentrations, canola and soybeans under the low inoculum concentration, and lupine and lentils under the high inoculum concentration (Figure 1).

## 4. Discussion

The host range of a pathogen can play an important role in designing effective disease management strategies [19]. In this study, *F. proliferatum* caused root rot in all crops examined. However, the soybeans, lupine, canola, and lentils displayed higher susceptibility, as indicated by symptom severity and reductions in emergences, plant heights, as well as root and shoot dry weights. In contrast, the barley and wheat, followed by the faba beans, showed greater tolerances to infection. Similar susceptibility trends were observed at both the seedling and adult plant stages across the crops. Other studies have reported similar phenomena, including reports with pea root rot caused by *Aphanomyces euteiches* [3], the dry root rot of chickpeas (*Cicer arietinum*) caused by *Rhizoctonia bataticola* [3,33], and the Fusarium root rot of soybeans [34] at various growth stages. These observations suggest that a tolerance to infection can persist in a host genotype, indicating the potential to mitigate disease pressure as plants mature. Conversely, susceptibility also continues into the later stages of growth.

Crop rotation is often regarded as a primary method for reducing pathogen inoculum, although its effectiveness depends on the availability of non-host crops [5,21,35]. The cross-pathogenicity of *Fusarium* spp. among pulse and cereal crops has been reported [36]. The fairly wide host range of *F. proliferatum* found in this study was not necessarily unexpected, given the reports of this pathogen being present on many host species [8,12,15,17,37]. However, the virulence observed on the soybeans, lupine, canola, lentils, and peas, and, to a lesser extent, on the barley, wheat, and faba beans, is concerning within a western Canadian context, since these represent most of the crops that can be grown in this region [16,38,39]. This suggests that rotation may have limited efficacy as a management strategy for the root rot caused by *F. proliferatum*, particularly since a rotation length of at least four years is recommended for most crops [21,40]. In addition to rotation and other cultural approaches, chemical treatments and biological control may contribute to the management of root rot [1,3,41,42]. Genetic resistance, however, can be one of the most effective approaches for plant disease control. 

Recent studies have indicated a high level of resistance in *Allium fistulosum* and *Allium schoenoprasum* accessions to isolates of *F. oxysporum* and *F. proliferatum* [43]. Resistance to Fusarium crown rot also has been widely reported in wheat and barley, in most cases being associated with non-pathogen-specific quantitative trait loci (QTLs) [44]. Similarly, QTLs associated with resistance to Fusarium root rot have also been identified in peas [45,46], as has a partial resistance or tolerance to root rot in soybeans [42]. There are limited reports, however, of a resistance or an enhanced tolerance in faba beans [47], canola [29], lentils [48], or lupine [49]. Nonetheless, the milder severity of root rot caused by *F. proliferatum* observed on barley and wheat in this study suggests that cereal crops may be less favorable hosts. In an earlier study, barley demonstrated a yield tolerance to the Fusarium crown rot caused by *Fusarium pseudograminearum* [50], while wheat genotypes with tolerances to Pythium root rot have also been reported [51]. A recent study from Kyrgyzstan suggested that *F. proliferatum* is non-pathogenic on wheat. [52]. While no oat genotypes were included in the present study, the response of this crop to *F. proliferatum* may be worth evaluating, as another report indicated that oats had greater tolerances than other cereal crops to the root and crown rot caused by *Fusarium* spp. [53,54]. The significant differences in root rot severity observed among cultivars of some species (faba beans, canola, and lentils) suggested diversity in the reaction to *F. proliferatum* within some crops, which may prove helpful in the development of root rot management plans. 

Molecular identification and phylogenetic analysis based on the translation elongation factor 1-alpha (TEF-1α) sequences, along with mating studies on multiple isolates from diverse hosts and locations, have suggested that there is no relation between *F. proliferatum* isolates and their hosts or geographic origins [18,37,55,56]. Nevertheless, an evaluation of vegetative compatibility among isolates of *F. proliferatum* indicated that isolates recovered from maize, onion, sugarcane, and rice could be classified into different vegetative compatibility groups (VCGs), indicating a correlation between VCGs and host preferences [12]. The fungal isolate used in the current study was obtained from canola [26], and assessments of the host responses to additional isolates collected from different species may be warranted to confirm the reactions observed here. Indeed, given that this isolate was selected as being the most aggressive among 20 isolates due to its virulence on the canola cv. ‘Westar’, the increased tolerance observed in the monocots of barley and wheat may also indicate a heightened level of adaptation of the isolate to a dicot host.

Principal component analysis has been recommended for the study of plant diseases given its ability to evaluate the relative importance of different variables in a quantitative manner [57]. For example, PCA has been used to assess leaf features, including color, shape, and texture, which could be used for disease identification [58], and for the evaluation of drought tolerance in canola based on morphological and agronomic traits [59]. In this study, the two principal components (PC1 and PC2) explained over 90% of the original variation among cultivars, indicating the capacity of these components to capture most of the diversity in responses to *F. proliferatum*. All five parameters used in the PCA were identified as major factors contributing to PC1. The PCA analysis highlighted the importance of considering multiple factors, including disease severity and reductions in growth parameters, for an improved assessment of host responses to the fungus. When only root rot severity was evaluated, all crops appeared to be susceptible to *F. proliferatum*, despite some variation in the disease ratings. However, the evaluation of the effect of the fungus on other parameters, such as emergence and biomass, highlighted the greater tolerance observed in some crops, particularly barley and wheat, relative to others. While disease severity is often used as the primary measure of fungal pathogenicity [60,61] or the host range [28], the effects of a pathogen on plant biomass, plant heights, and seedling emergences have also been explored in several studies [34,41,62]. Notable reductions in dry weight (>50%) were documented for *Allium* spp. inoculated with *F. proliferatum*, reflecting severe root rot [63]. To the best of our knowledge, this study is the first to apply PCA for the evaluation of the utility of different plant-related parameters for host range identification in *F. proliferatum*.

This study indicated that barley, wheat, and, to a lesser extent, faba beans showed a degree of tolerance to *F. proliferatum*, whereas canola and other legume crops exhibited greater susceptibility. As such, crop rotations involving barley, wheat, or faba beans, with appropriate intervals, may help to alleviate disease pressure. Additionally, while there was variability in the tolerances observed among the cultivars of these species, none of the crops were fully resistant. Given the importance of these crops in western Canadian agriculture, rotations alone may not fully mitigate Fusarium root rot under high disease pressure. Integrating additional disease control strategies may be necessary for effective management. Furthermore, due to the limitations in the number of fungal isolates and crop cultivars examined in this study, future research should encompass a broader range of isolates from various hosts to assess their cross-pathogenicity. Screening for resistances in crops that have exhibited variations in their responses to inoculation is essential for identifying resistant germplasms.

## Figures and Tables

**Table 1:** The root rot severity of eight crop species at 21 days after seeding in a potting medium treated with different concentrations of *Fusarium proliferatum* inoculum.

Treatment *^a^*	Root Rot Severity *^b^*							
	Wheat	Barley	Faba Bean	Pea	Lentil	Canola	Lupine	Soybean
Control	0.00 a, A	0.00 a, A	0.00 a, A	0.00 a, A	0.00 a, A	0.00 a, A	0.00 a, A	0.00 a, A
Low	1.29 b, A	1.47 b, AB	1.77 b, BC	1.97 b, C	1.96 b, C	2.66 b, D	2.87 b, DE	3.03 b, E
High	2.12 c, A	1.98 c, A	2.51 c, B	2.73 c, B	3.35 c, C	3.38 c, C	3.33 c, C	3.42 c, C

*^a^* Control, non-inoculated; Low, treated with a low concentration (3 × 10^4^ colony-forming units (cfu)/g potting medium) of *F. proliferatum* inoculum; High, treated with a high concentration (6 × 10^4^ cfu/g potting medium) of *F. proliferatum* inoculum. The results for each plant species represent the averages from 2–4 cultivars of each crop (see Supplementary Table S1 for the data for the individual cultivars). *^b^* Root rot disease severity as assessed on a 0–4 scale [25], where 0 = healthy roots; 1 = small, light-brown lesions are present on <25% of the tap root; 2 = brown lesions are present on 25–49% of the tap root; 3 = brown lesions are present on 50–74% of the tap root, with the tap root constricted; and 4 = the tap root is severely girdled, brown lesions are present on >75% of the tap root with limited lateral roots. Note: Different lowercase letters indicate significant differences (*p* < 0.05) within columns, while different uppercase letters indicate significant differences within rows.

**Table 2:** Average plant heights, seedling emergences, and shoot and root weights of the eight crop species at various times after seeding in a potting medium treated with different concentrations of *Fusarium proliferatum* inoculum.

Treatment *^a^*	Plant Height (cm) *^b^*							
	Wheat	Barley	Faba Bean	Pea	Lentil	Canola	Lupine	Soybean
Control	18.31 a	17.19 a	10.14 a	15.66 a	11.33 a	3.73 a	12.52 a	6.76 a
Low	17.27 a	17.21 a	7.79 b	8.13 b	4.34 b	1.83 b	5.62 b	2.66 b
High	12.01 b	10.87 b	6.86 b	5.59 c	2.16 c	1.34 b	3.60 c	1.81 b
	**Seedling Emergence *^c^***							
	**Wheat**	**Barley**	**Faba Bean**	**Pea**	**Lentil**	**Canola**	**Lupine**	**Soybean**
Control	9.57 a	9.80 a	6.55 a	9.38 a	9.35 a	9.40 a	9.90 a	8.70 a
Low	8.80 b	9.15 a	6.70 a	5.85 b	3.80 b	2.55 b	4.75 b	1.53 b
High	6.53 c	7.10 b	4.75 b	2.78 c	0.65 c	0.45 c	1.85 c	0.53 c
	**Shoot Dry Weight (g) *^d^***							
	**Wheat**	**Barley**	**Faba Bean**	**Pea**	**Lentil**	**Canola**	**Lupine**	**Soybean**
Control	0.42 ab	0.45 a	1.95 a	1.14 a	0.56 a	0.35 a	1.49 a	1.24 a
Low	0.45 b	0.50 a	1.68 b	0.67 b	0.12 b	0.18 ab	0.61 b	0.39 b
High	0.29 a	0.36 a	1.42 c	0.27 c	0.02 b	0.04 b	0.18 c	0.25 b
	**Root Dry Weight (g) *^e^***							
	**Wheat**	**Barley**	**Faba Bean**	**Pea**	**Lentil**	**Canola**	**Lupine**	**Soybean**
Control	0.55 a	0.54 a	0.87 a	0.69 a	0.30 a	0.07 a	0.33 a	0.32 a
Low	0.37 b	0.46 a	0.74 b	0.53 b	0.07 b	0.05 a	0.11 b	0.08 b
High	0.19 c	0.22 b	0.71 b	0.19 c	0.02 b	0.01 a	0.04 b	0.06 b

*^a^* Control, non-inoculated; Low, treated with a low concentration (3 × 10^4^ colony-forming units (cfu)/g potting medium) of *F. proliferatum* inoculum; High, treated with a high concentration (6 × 10^4^ cfu/g potting medium) of *F. proliferatum* inoculum. The results for each plant species represent the averages from 2–4 cultivars of each crop (see Supplementary Table S1 for the data for the individual cultivars). *^b^* Plant height was measured at 14 days after seeding. *^c^* Seedling emergence (the average number of plants per experimental unit (cup)) was measured at 7 days after seeding. *^d^* Shoot dry weight was measured at 21 days after seeding. *^e^* Root dry weight was measured at 21 days after seeding. Note: Different lowercase letters indicate significant differences (*p* < 0.05) within columns.

**Table 3:** A comparison of the root rot severity at maturity (after flowering) on the nine cultivars representing seven different crop species grown in a potting medium inoculated with *Fusarium proliferatum*.

Crop	Cultivar	Disease Severity *^a^*
Barley	Canmore	1.08 a
Wheat	AC Crystal	1.15 a
Faba bean	Fabelle	2.00 b
Pea	AAC Carver	2.62 c
Canola	Westar	3.00 d
Soybean	AKRAS R2	3.06 d
Pea	CDC Amarillo	3.20 d
Pea	CDC Greenwater	3.26 d
Lupine	Arabella	3.57 e

*^a^* Root rot disease severity as assessed on a 0–4 scale [25], where: 0 = healthy roots; 1 = small, light-brown lesions are present on <25% of the tap root; 2 = brown lesions are present on 25–49% of the tap root; 3 = brown lesions are present on 50–74% of the tap root, with the tap root constricted; and 4 = the tap root is severely girdled, brown lesions are present on >75% of the tap root with limited lateral roots. Disease assessments were conducted following the completion of flowering for each cultivar/crop. Note: Different lowercase letters indicate significant differences (*p* < 0.05) within columns.

## Data Availability

The data presented in this study are available from the corresponding authors upon request.

## Supplementary Materials

The following supporting information can be downloaded at: https://www.mdpi.com/article/10.3390/pathogens13050407/s1, Figure S1: Root rot disease rating scale for eight crop species; Figure S2: Reductions (%), relative to non-inoculated controls, in plant height, seedling emergence, and shoot and root weights of 20 cultivars representing eight crop species grown in a potting medium treated with low (a, c, e, g) or high (b, d, f, h) concentrations of *Fusarium proliferatum* (3 × 10^4^ and 6 × 10^4^ colony-forming units/g potting medium, respectively); Figure S3: Detection of *Fusarium proliferatum* in plant root tissues via PCR analysis with the *F. proliferatum*-specific primers TH5-F/TH6-R; Table S1: Comparison of root rot severity on cultivars representing eight different crop species at 21 days after seeding in a potting medium treated with different concentrations of *Fusarium proliferatum* inoculum.; Table S2: Principal component analysis of root rot disease severity and reductions in emergence, plant height, shoot and root dry weights of 20 cultivars representing 8 crop species grown in a potting medium treated with different concentrations of *Fusarium proliferatum* inoculum.
