# Supplementary material for: The Host Range of Fusarium proliferatum in Western Canada
Source: Pathogens. 2024 May 14;13(5):407. doi: 10.3390/pathogens13050407 (PMC11123688; doi:10.3390/pathogens13050407)
Supplement: Supplementary file 1 [file pathogens-13-00407-s001.zip › pathogens-2928774-supplementary.pdf]

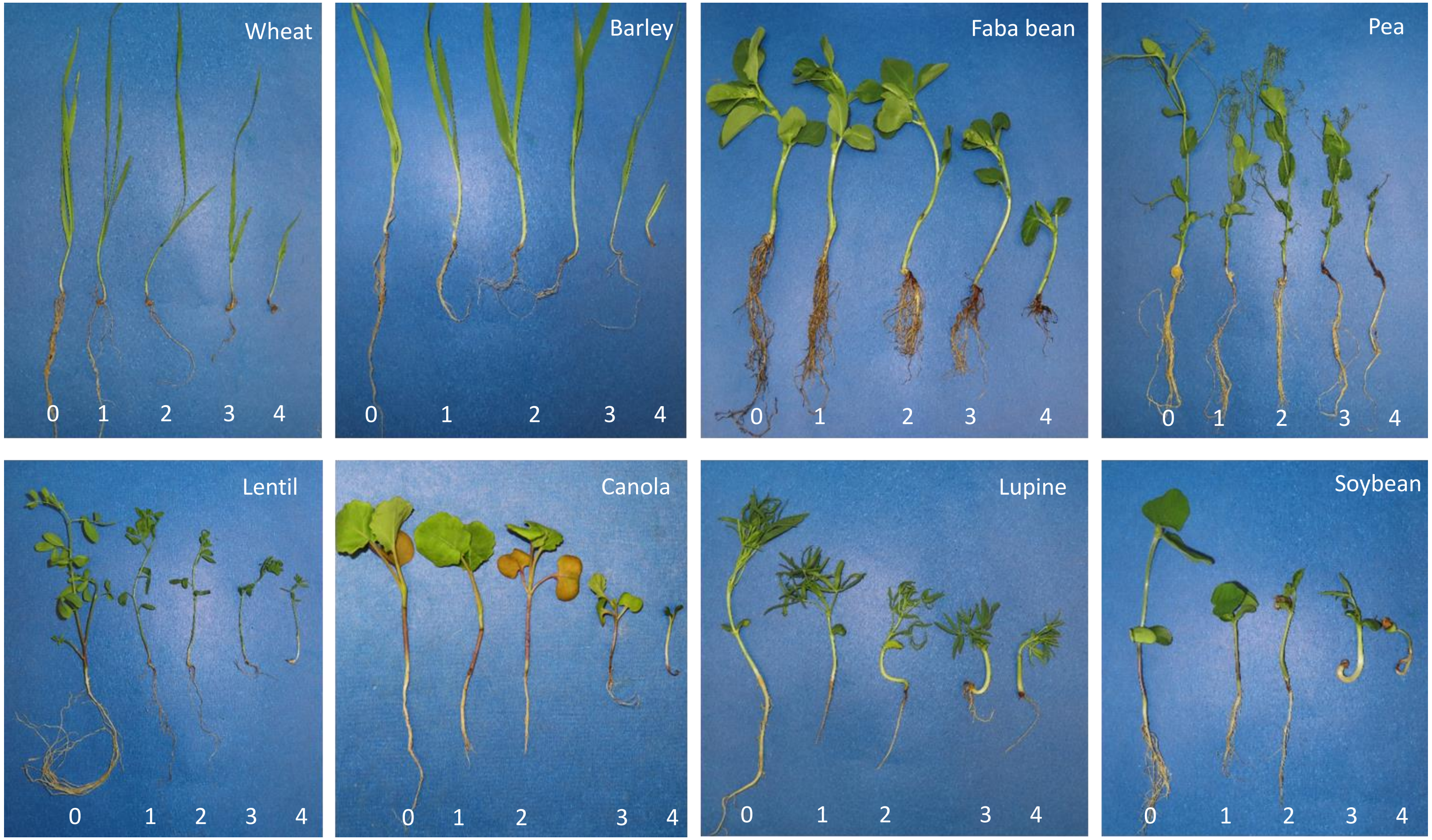


**Supplementary Figure S1**. Root rot disease rating scale for eight crop species, where: 0 = healthy roots; 1 = small, light-brown lesions on < 25% of the tap root; 2 = brown lesions on 25-49% of the tap root; 3 = brown lesions on 50-74% of the tap root, tap root constricted; and 4 = tap root severely girdled, brown lesions on > 75% of the tap root with limited lateral roots. The scale was adapted from Hwang et al. [25].

**Supplementary Table S1.** Comparison of root rot severity on cultivars representing eight different crop species at 21 days after seeding in potting medium treated with different concentrations of *Fusarium proliferatum* inoculum.

| Crop | Cultivar | Disease Severity[a] | |
|---|---|---|---|
| | | Low Inoculum[b] | High Inoculum[c] |
| Wheat | AC Crystal | 1.06 a | 2.29 AB |
| | Katepwa | 1.46 ab | 2.00 A |
| | Lillian | 1.35 ab | 2.06 AB |
| Barley | AB Tofield | 1.23 ab | 1.86 A |
| | Canmore | 1.72 bcd | 2.10 AB |
| Faba bean | Malik | 1.31 ab | 2.13 AB |
| | Fabelle | 2.24 de | 2.89 CDE |
| Pea | CDC Amarillo | 1.57 abc | 2.56 BCD |
| | AAC Barrhead | 2.01 cde | 2.37 ABC |
| | CDC Greenwater | 2.03 cde | 2.90 CDE |
| | AAC Carver | 2.26 e | 3.10 EFG |
| Lentil | CDC Nimble | 1.62 bc | 3.01 DEF |
| | CDC Lima CL | 2.30 ef | 3.70 H |
| Canola | Westar | 2.33 ef | 3.06 DEF |
| | L255PC | 2.99 g | 3.68 H |
| Lupine | Arabella | 2.83 fg | 2.97 DE |
| | Mirabor | 2.90 g | 3.68 H |
| Soybean | AAC Mandor | 2.51 efg | 3.13 EFG |
| | AKRAS R2 | 3.03 gh | 3.53 FGH |
| | OT15-02 | 3.55 h | 3.61 GH |

[a] Root rot disease severity as assessed on a 0-4 scale [25], where: 0 = healthy roots; 1 = small, light-brown lesions on < 25% of the tap root; 2 = brown lesions on 25-49% of the tap root; 3 = brown lesions on 50-74% of the tap root, tap root constricted; and 4 = tap root severely girdled, brown lesions on > 75% of the tap root with limited lateral roots.

[b] Treated with a low concentration ($3 \times 10^4$ colony forming units (cfu)/g potting medium) of *F. proliferatum* inoculum.

[c] Treated with a high concentration ($6 \times 10^4$ cfu/g potting medium) of *F. proliferatum* inoculum.

Note: Different lowercase letters indicate significant differences ($p < 0.05$) within the 'Low Inoculum' column, while different uppercase letters indicate significant differences within the 'High Inoculum' column.

**Supplementary Table S2.** Principal Component Analysis of root rot disease severity and reductions in emergence, plant height, shoot and root dry weights of 20 cultivars representing 8 crop species grown in potting medium treated with different concentrations of *Fusarium proliferatum* inoculum.

| Parameter | Low Inoculum Concentration[a] | | | | | High Inoculum Concentration[b] | | | | |
|---|---|---|---|---|---|---|---|---|---|---|
| | PC1 | PC2 | PC3 | PC4 | PC5 | PC1 | PC2 | PC3 | PC4 | PC5 |
| Disease Severity | -0.4254 | -0.5600 | 0.6152 | -0.3559 | -0.0247 | -0.4364 | -0.5245 | -0.7019 | 0.0610 | -0.1954 |
| Emergence Reduction | -0.4645 | -0.0237 | 0.1613 | 0.8704 | 0.0129 | -0.4695 | -0.0444 | 0.1828 | 0.5337 | 0.6778 |
| Plant Height Reduction | -0.4613 | -0.1467 | -0.5582 | -0.1369 | -0.6598 | -0.4657 | -0.0640 | 0.1921 | -0.8192 | 0.2664 |
| Shoot Dry Weight Reduction | -0.4707 | -0.0379 | -0.4358 | -0.1826 | 0.7442 | -0.4665 | -0.1095 | 0.5672 | 0.1987 | -0.6397 |
| Root Dry Weight Reduction | -0.4110 | 0.8143 | 0.3067 | -0.2525 | -0.1008 | -0.3932 | 0.8408 | -0.3397 | 0.0297 | -0.1491 |

[a] Low, treated with a low concentration ($3 \times 10^4$ colony forming units (cfu)/g potting medium) of *F. proliferatum* inoculum; [b]High, treated with a high concentration ($6 \times 10^4$ cfu/g potting medium) of *F. proliferatum* inoculum.

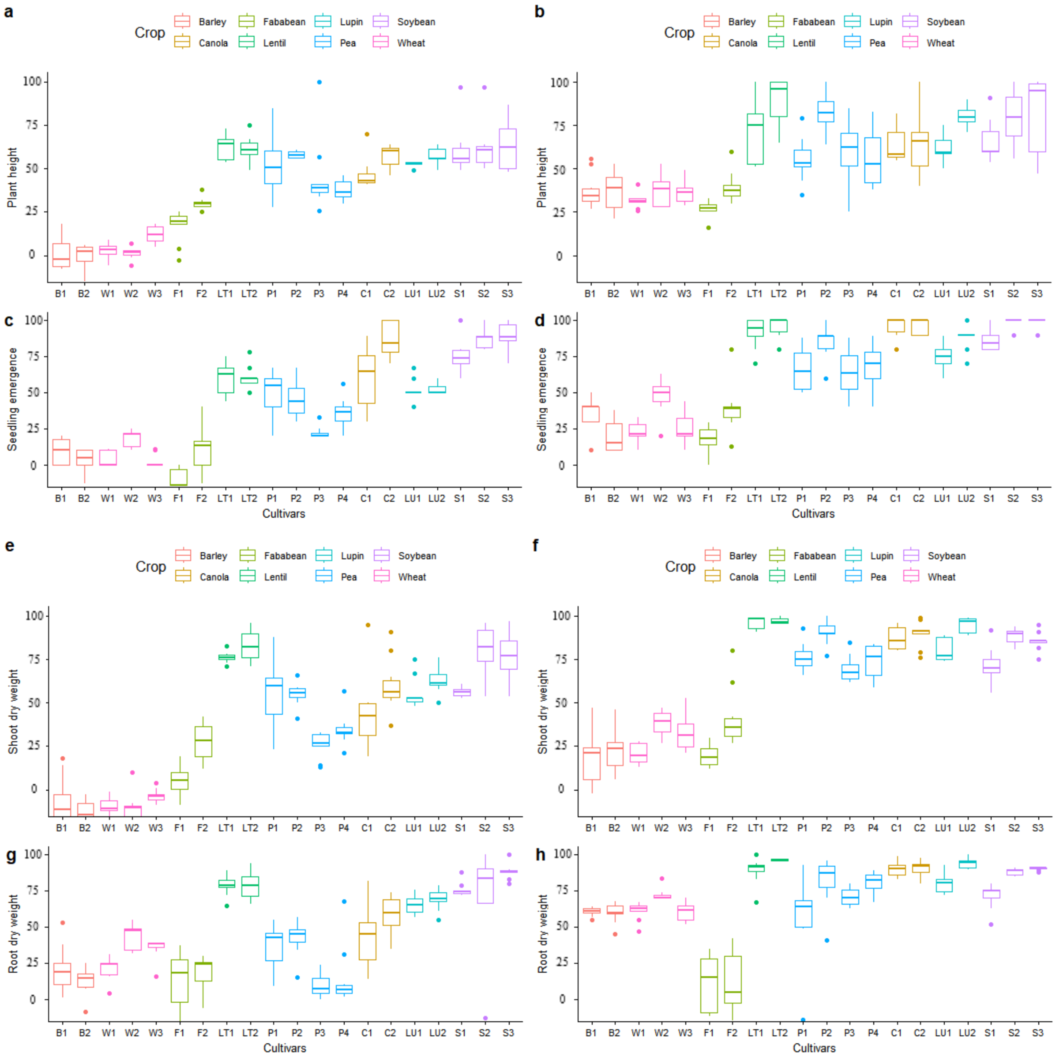


**Supplementary Figure S2.** Reductions (%), relative to non-inoculated controls, in plant height, seedling emergence, and shoot and root weights of 20 cultivars representing eight crop species grown in potting medium treated with low (a, c, e, g) or high (b, d, f, h) concentrations of *Fusarium proliferatum* ($3 \times 10^4$ and $6 \times 10^4$ colony forming units/g potting medium, respectively). Plant height (a,b) was measured at 14 days after seeding, seedling emergence (c,d) was measured at 7 days after seeding, shoot dry weight (e,f) was measured at 21 days after seeding, and root dry weight (g,h) was measured at 21 days after seeding. B1, barley cultivar ‘AB Tofield’; B2, barley ‘Canmore’; W1, wheat ‘Katepwa’; W2, wheat ‘AC Crystal’; W3, wheat ‘Lillian’; P1, pea ‘CDC Greenwater’; P2, pea ‘AAC Carver’; P3, pea ‘CDC Amarillo’; P4, pea ‘AAC Barrhead’; S1, soybean ‘AAC Mandor’; S2, soybean ‘OT15-02’; S3, soybean ‘AKRAS R2’; LU1, lupine ‘Arabella’; LU2, lupine ‘Mirabor’; L1, lentil ‘CDC Nimble’; L2, lentil ‘CDC Lima CL’; F1, faba bean ‘Malik’; F2, faba bean ‘Fabelle’; C1, canola ‘Westar’; C2, canola ‘L255PC’.

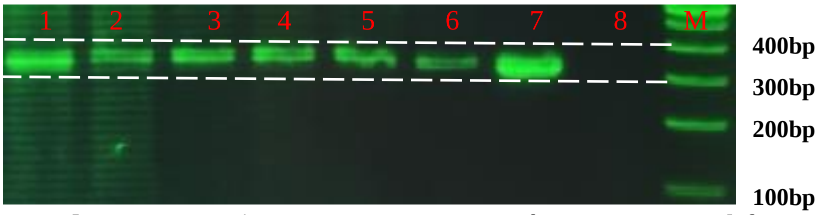

**Supplementary Figure S3.** Detection of *Fusarium proliferatum* in plant root tissues by PCR analysis with the *F. proliferatum*-specific primers TH5-F/TH6-R [31]. Plants were grown in potting medium inoculated with the fungus (3 × $10^4$ cfu/g potting medium) and root tissues were collected shortly after flowering. Total genomic DNA was extracted and subjected to PCR. Lane 1, pea cultivar ‘CDC Greenwater’; lane 2, pea ‘AAC Carver’; lane 3, faba bean ‘Fabelle’; lane 4, soybean ‘AKRAS R2’; lane 5, lupine ‘Arabella; lane 6, wheat ‘AC Crystal’; lane 7, *F. proliferatum* isolate P002 (positive control); lane 8, nuclease-free water (negative control); lane M, 100 bp DNA ladder (Thermo Fisher Scientific, Mississauga, ON).
